# Homocysteine combined with multi-index screening for pregnancy complications: a narrative review

**DOI:** 10.3389/fendo.2026.1835074

**Published:** 2026-05-19

**Authors:** Chunli An, Yan Tang, Chunli Yin, Min Wang, Guihua Zuo

**Affiliations:** Department of Obstetrics, Anyue Maternal and Child Health Hospital, Ziyang, China

**Keywords:** homocysteine, multi-marker screening, one-carbon metabolism, pregnancy complications, risk assessment

## Abstract

Employing a narrative review approach, this article examines the value of combining homocysteine (Hcy) with multiple indicators for screening pregnancy complications. Hcy serves as a key biomarker in one-carbon metabolism and vascular endothelial function. Elevated Hcy levels are significantly correlated with adverse pregnancy outcomes, including preeclampsia (PE), fetal growth restriction (FGR), gestational diabetes mellitus (GDM), and intrahepatic cholestasis of pregnancy (ICP). Hcy levels are influenced by nutritional factors (folate, vitamin B12) and genetic factors (MTHFR polymorphisms). However, the predictive power of Hcy alone is limited due to variations in gestational age, ethnicity, nutritional status and genetic background. Recent studies have therefore explored combined screening strategies: Hcy together with platelet parameters helps differentiate the risk of ICP from that of GDM; adding uterine artery (UtA) Doppler ultrasonography improves the diagnostic sensitivity for detecting PE and FGR; integrating glycolipid metabolic markers (Glycosylated Serum Protein, Cystatin-C, apoB/apoA1) provides additional predictive value for adverse outcomes in GDM. Positioning Hcy as a functional endpoint of gene-nutrient interactions and combining it with nutritional and genetic assessments may facilitate early warning of pregnancy complications and enable individualized intervention.

## 1 Introduction

Pregnancy complications—including pre-eclampsia (PE), gestational diabetes mellitus (GDM), fetal growth restriction (FGR), and preterm delivery (PTD) —are leading causes of adverse maternal and fetal outcomes according to the World Health Organization (WHO) (https://www.who.int/publications/i/item/WHO-RHR-18.14). Early identification of high-risk pregnant women facilitates timely clinical management and targeted interventions, thereby improving perinatal prognosis. However, current screening methods rely on single or limited indicators, such as maternal urine tests, peripheral blood markers, uterine artery pulsatility index, and mean arterial pressure, which exhibit limited predictive power (1, 2). Consequently, there is an urgent need for more accurate early risk assessment tools to enhance the prediction, prevention, and treatment of pregnancy complications (3).

Homocysteine (Hcy) is a sulfur-containing, non-protein amino acid derived from the demethylation of dietary methionine (4). Its metabolism depends on folic acid, vitamin B12, and vitamin B6 as coenzymes, and is tightly regulated by key enzymes, particularly methylenetetrahydrofolate reductase (MTHFR) (5). Disruption of this metabolic balance due to genetic factors or nutritional deficiencies can lead to hyperhomocysteinemia (HHcy) (6). Characterized by abnormally elevated blood Hcy levels, HHcy is widely recognized as an independent risk factor for various vascular diseases. Its pathogenic mechanisms include inducing vascular endothelial dysfunction, exacerbating oxidative stress, and promoting a prothrombotic state, all of which contribute to the pathogenesis of cardiovascular and other vascular disorders (7).

**Figure 1:**
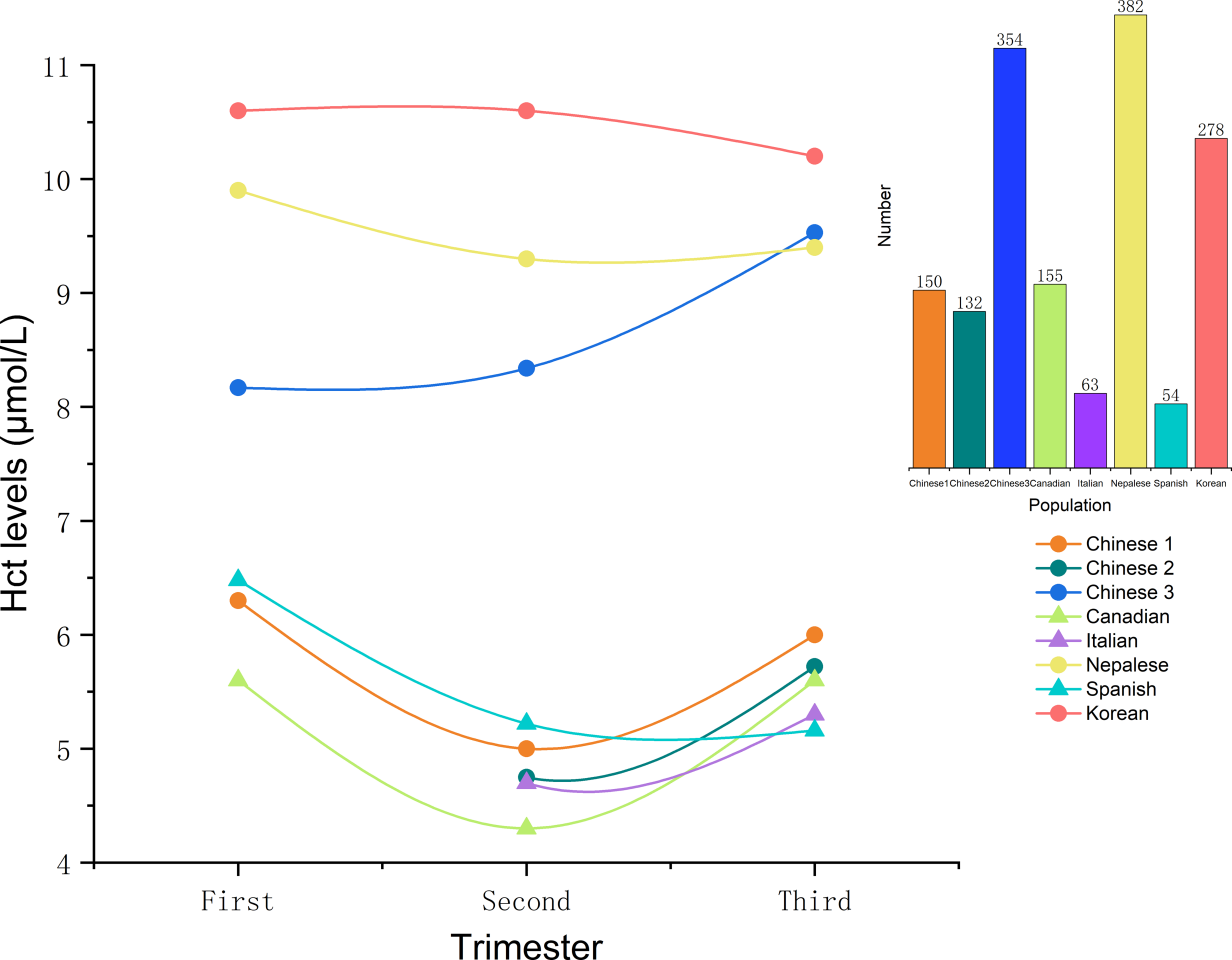
Homocysteine levels at different stages of normal pregnancy from research studies in various countries (6, 10, 14, 15). The line graph depicts homocysteine levels throughout normal pregnancy as reported in studies from various countries; circles indicate the median, and triangles represent the mean. The bar graph shows the number of participants included in each respective study. Figure was plotted using Origin 2021.

Recent research has expanded the scope of Hcy to include gestational and postpartum health (8). Hcy levels are readily measurable in maternal peripheral blood. However, the lack of uniform reference ranges across laboratories complicates the diagnosis of HHcy. In the non-pregnant population, levels below 15 μmol/L are generally considered normal, though some studies adopt 13 or 14 μmol/L as the upper limit. Levels of 15~30 μmol/L indicate mild elevation, 30~60 μmol/L moderate elevation, and >60 μmol/L severe elevation (5). During pregnancy, plasma Hcy levels are typically lower than in non-pregnant women and follow a “U-shaped” curve: decreasing in the first trimester, reaching a nadir in the second, and gradually rising in the third as shown in **Figure 1** (9). This pattern is likely attributable to hemodilution from increased plasma volume, enhanced glomerular filtration, and fetal uptake of Hcy (10).

Existing evidence suggests that elevated Hcy levels during pregnancy are associated with a range of adverse outcomes. In normal pregnancies, maternal Hcy concentrations range from 5.43 to 9.42 μmol/L, with a gradual decline in the second and third trimesters. When Hcy exceeds a threshold of 7.7–8 μmol/L, the risks of early spontaneous miscarriage, preeclampsia, and fetal growth restriction increase significantly. If Hcy rises above 15 μmol/L, the risk of placental abruption is elevated more than 5 fold (11). Evidence from the first trimester (weeks 10–14) further confirms that high Hcy levels at this time point correlate strongly with PE, FGR, placental abruption, and recurrent miscarriage, and that these associations follow a dose−response pattern with disease severity (12). Nevertheless, the links between Hcy and both placental abruption and gestational diabetes mellitus remain debated, as some studies have failed to replicate these findings. This inconsistency may stem from differences in study populations, timing of Hcy measurement, and heterogeneity in diagnostic criteria (10, 13). Mechanistic studies suggest that Hcy may contribute to adverse pregnancy outcomes through pathways such as vascular endothelial injury and oxidative stress. Whether folic acid supplementation serves as an effective intervention—and if so, the optimal timing for its administration—still requires validation through high−quality research (10).

Most existing studies have focused on Hcy as a single biomarker. However, the predictive performance of a single indicator is vulnerable to individual variability and confounding factors (14).The pathophysiology of pregnancy complications is inherently complex, involving multiple interacting pathways such as coagulation abnormalities, vascular endothelial injury, oxidative stress imbalance, and metabolic disturbances. Relying on any single biomarker is unlikely to fully capture the underlying pathological changes.

In recent years, research on combining multiple indicators to predict the risk of pregnancy complications has steadily grown. Adopting the concept of combined screening, this review synthesizes evidence from the past decade, summarizes the advances and clinical utility of Hcy−based multi−marker panels for predicting pregnancy complications, and aims to inform risk management during pregnancy in clinical practice.

## 2 Combined application of Hcy with other indicators

**Figure 2:**
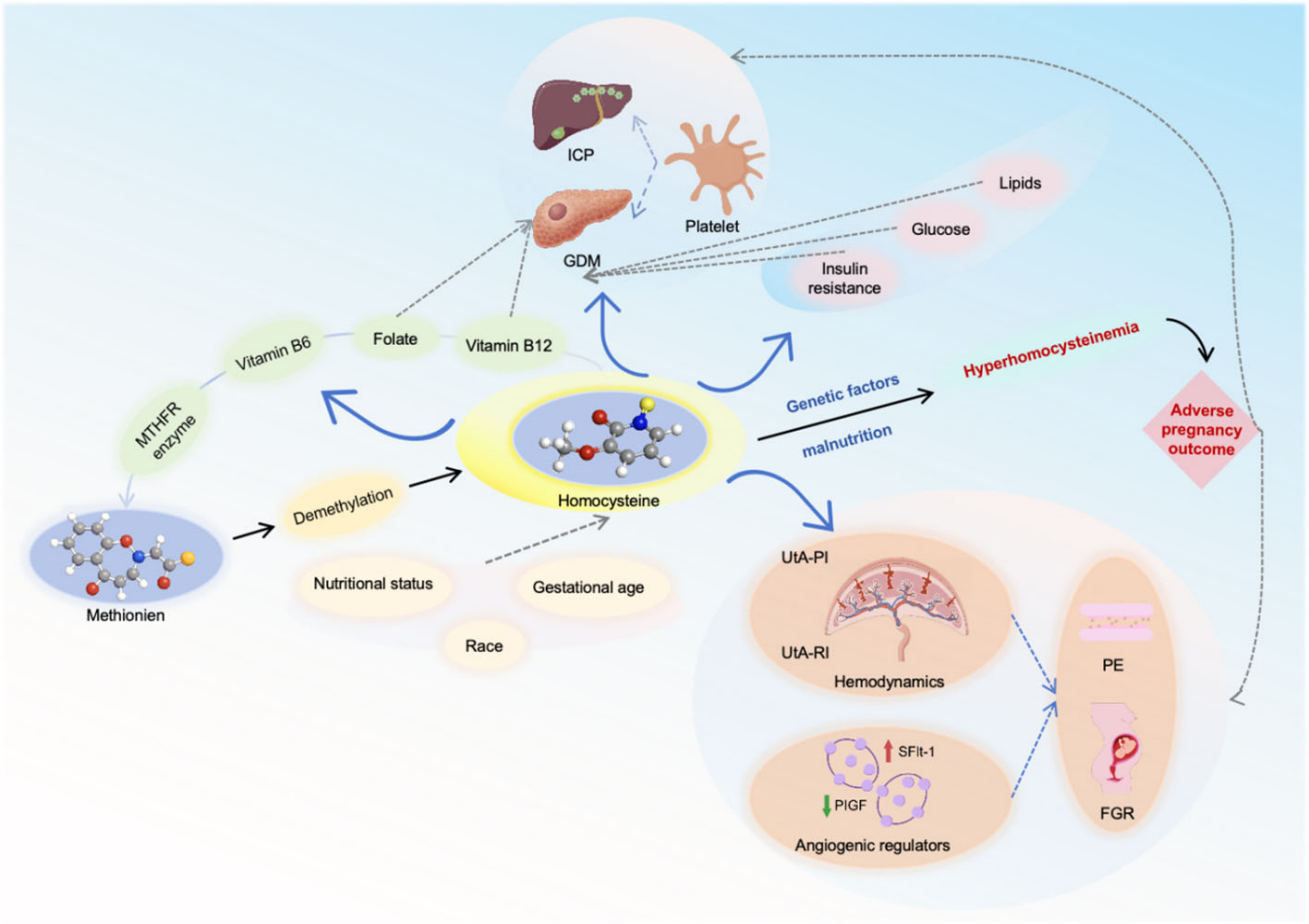
Combined prediction of hcy with other biomarkers. FGR, fetal growth restriction; GDM, gestational diabetes mellitus; ICP, intrahepatic cholestasis of pregnancy; MTHFR, methylenetetrahydrofolate reductase; pe, preeclampsia; PlGF, placental growth factor; Sflt-1, soluble Fms-like tyrosine kinase-1; UTA-PI, uterine artery pulsatility index; UtA-RI, uterine artery resistance index. The icons in the image are sourced from CNSknowallplatform (https://cnsknowall.com).

Incorporating Hcy into a multi−marker screening panel may help capture the complex pathophysiology of pregnancy complications more comprehensively. As shown in **Figure 2** and **Table 1**, the synergy and complementarity of multi−dimensional indicators, as opposed to a single−biomarker approach, could enhance predictive performance for disease outcomes. Nevertheless, further validation through high−quality studies remains necessary.

**Table 1:** Summary of studies combining Hcy with other indicators for screening pregnancy complications.

Author (year)	Country	Study design	Population (case/control)	Trimester	Hcy level (μmol/L)*	Combined indicator(s)	Complications	Key findings	Key limitations (simplified)
Yu et al. (27)	China	Retrospective cohort	201 HHcy/11,352 normal	Third	> 15 (cutoff)	Platelet count (Q1–Q4)	ICP, GDM	Hcy+low PC → ↑ ICP risk; Hcy+high PC → ↓ GDM risk	Single-center; retrospective bias; causality unclear
Maged et al. (35)	Egypt	Prospective cohort	PE (n=45), IUGR (n=52), other (n=59)/uncomplicated(n=297)	Second (15–19w)	Uncomplicated: 4.70; PE: 7.03; IUGR: 6.32; Other: 6.60	UtA-RI	PE, FGR	Hcy+UtA-RI → significant predictor of placenta-derived complications	No continuous follow-up of neonatal/maternal outcomes
Ramesh et al. (36)	India	Prospective cohort	HHcy pregnant/normal pregnant(n=100)	Second (18–28w)	≥ 9.7 (95th centile)	UtA-PI (>90th centile)	FGR, preterm birth, low Apgar	High specificity, low sensitivity for FGR, preterm, low Apgar; no significant prediction for hypertension or NICU admission	Small sample size; only UtA-PI used (no RI or notching)
Su et al. (40)	China	Retrospective case-control	54 GDM/54 controls	Not specified	GDM: 11.41 ± 2.48; Control: 5.91 ± 1.81	FPG, HbA1c, FINS, HOMA-IR, cystatin C	GDM	Hcy positively correlated with all above indicators	Retrospective; single-center; no adjustment for BMI, folate, or B12; single Hcy measurement
Chen et al. (52)	China	Retrospective cohort	119 GDM/128 controls	Third	GDM: 21 (18–24); Control: 12 (11–14)	GSP, cystatin C	GDM	Higher GSP, Hcy, cystatin C → higher risk of adverse outcomes	Small sample; single-center; only three markers studied
Li et al. (42)	China	Prospective cohort	150 diet-controlled GDM/150 NGT	Third	GDM: 6.30 (5.48–8.00); NGT: 4.05 (3.10–5.40)	FPG, apoB/apoA1 ratio	GDM	Higher Hcy, FPG, apoB/apoA1 in GDM; correlation with worse perinatal outcomes	Single-center; only diet-controlled GDM; single Hcy measurement; no folate/B12 data

*Hcy levels presented as mean ± SD or median (IQR) as reported. apoB/apoA1, apolipoprotein B/apolipoprotein A1 ratio; FGR, fetal growth restriction; FINS, fasting insulin; FPG, fasting plasma glucose; GDM, gestational diabetes mellitus; GSP, glycated serum protein; HbA1c, glycated hemoglobin; Hcy, homocysteine; HHcy, hyperhomocysteinemia; HOMA-IR, homeostatic model assessment for insulin resistance; ICP, intrahepatic cholestasis of pregnancy; IUGR, intrauterine growth restriction; NGT, normal glucose tolerance; NICU, neonatal intensive care unit; PC, platelet count; PE, preeclampsia; UtA-PI, uterine artery pulsatility index; UtA-RI, uterine artery resistance index.

### 2.1 Hcy combined with platelet parameters

Platelets play a pivotal role in vascular endothelial injury and coagulation regulation (16). For instance, some studies have reported that an elevated platelet count (PC) independently predicts GDM (17, 18), whereas a decreased PC independently predicts PE (19, 20). Other studies have only observed between−group differences in PC levels in GDM or PE groups, with the independent association disappearing after adjusting for confounders (21–23). Still other studies have found no significant association between PC levels and either GDM or PE;Mean platelet volume (MPV) is considered a promising biomarker. In conditions such as PE and GDM, which are characterized by inflammation and endothelial dysfunction, MPV values are generally elevated (24). Notably, after 20 weeks of gestation, MPV shows good diagnostic predictive value for PE (25). In addition, platelet distribution width (PDW) is significantly increased in women with unexplained recurrent pregnancy loss (URPL), suggesting that it may serve as a predictive marker for URPL (26).

Given the considerable heterogeneity among studies on single platelet parameters, combining them with other biomarkers that reflect vascular endothelial injury and metabolic status may improve the risk assessment efficacy for pregnancy complications. A large-scale retrospective cohort study involving 11553 pregnant women in Changzhou, China, provided evidence supporting the clinical utility of combining Hcy with platelet parameters in gestational risk assessment (27). The study found that elevated Hcy combined with low PC before delivery was associated with an increased risk of intrahepatic cholestasis of pregnancy (ICP). The proposed mechanism involves HHcy-induced endothelial damage and subsequent platelet consumption, which may exacerbate hepatic microcirculatory disturbances and bile acid metabolism abnormalities. Interestingly, a different pattern emerged for GDM: pregnant women with high Hcy levels and elevated PC showed a reduced risk of developing GDM. This paradoxical finding suggests that in the absence of significant endothelial injury and platelet consumption, elevated Hcy may reflect nutritional or genetic predisposition rather than severe vascular pathology. In this context, platelets may act as an “effect modifier,” modulating the relationship between Hcy and disease outcomes. Beyond platelet count, other platelet parameters such as mean platelet volume (MPV) and platelet distribution width (PDW) have also garnered research interest. For instance, patients with ICP exhibit significantly elevated MPV levels compared to those without ICP, suggesting that platelet activation status may contribute to disease pathogenesis. Although research in this area remains limited, the screening strategy combining Hcy with platelet parameters offers a novel and cost-effective approach for the clinical prevention and management of ICP and GDM, thereby helping to advance the screening and management of pregnancy complications.

### 2.2 Hcy combined with uterine artery hemodynamics

Given that HHcy-induced vascular endothelial damage is a core mechanism underlying PE and FGR, combining Hcy with indicators of vascular function and placental perfusion offers a rational strategy to improve risk prediction for these conditions (28, 29).

Uterine artery (UtA) Doppler ultrasound is a convenient, non-invasive screening tool. Using parameters such as the pulsatility index (PI), resistance index (RI), and early diastolic notch (notch), it is widely employed to assess utero-placental hemodynamics and identify pregnancies at risk of placental dysfunction (30–33). Among these, RI demonstrates the best overall performance (AUC 0.91, sensitivity 73%, specificity 90%), followed by PI and notching, with sensitivities of 65% and 54%, respectively (31). Notably, even the best-performing single indicator (RI) still has considerable room for improvement in sensitivity. UtA Doppler primarily reflects the functional status of placental perfusion resistance, whereas hyperhomocysteinemia (HHcy) contributes to the pathogenesis of placental dysfunction through vascular endothelial injury (34).Combining the two approaches may offer complementarity from functional and biochemical dimensions, thereby compensating for the limited sensitivity of any single indicator and improving the early predictive accuracy for placenta−derived complications such as preeclampsia and fetal growth restriction.

A prospective cohort study of 453 pregnant women to evaluate the predictive value of combined screening (35). Hcy levels were measured at 15–19 weeks of gestation, and UtA Doppler ultrasound was performed at 18–22 weeks. Women who subsequently developed PE, FGR, or other placenta-related complications had significantly higher Hcy levels and UtA-RI compared to the control group. Combining Hcy with UtA indices improved the predictive sensitivity for PE to 85.2%, substantially higher than that of Hcy alone (73.3%) or UtA alone (60.0%). These findings suggest that the combined strategy has significant predictive value for placenta-derived complications such as PE and FGR. However, the predictive performance of combined screening is not consistent across all settings. A prospective cohort study from India reported that combining second-trimester maternal serum Hcy (threshold ≥9.7 μmol/L) with UtA-PI achieved high specificity for predicting FGR (98.9%) and spontaneous preterm birth (83.4%, p<0.01) (36). This indicates that when both markers are abnormal, the combination effectively identifies high-risk populations. However, the sensitivity was low (e.g., only 14.3% for FGR), and the combined strategy was not superior to single markers for predicting gestational hypertension. These findings suggest that Hcy-UtA combined screening may be more suitable for “high-risk confirmation” than for universal “broad screening,” particularly for severe placenta-derived complications like FGR. Thus, the clinical utility of this combined approach should be evaluated on a case-by-case basis according to the specific setting, outcomes of interest, and population features. Its application in the general population warrants caution to prevent underdiagnosis.

### 2.3 Hcy combined with glucose and lipid metabolism indicators

In recent years, various biomarkers related to glycolipid metabolism and inflammatory responses have been explored for the early prediction of gestational diabetes mellitus (GDM), yet their diagnostic efficacy when used alone remains generally limited. A prospective diagnostic accuracy study from Turkey, which included 250 pregnant women, assessed this issue. In that study, multiple candidate indicators, including the Lymphocyte-to-High-Density Lipoprotein Cholesterol Ratio(LHR), Monocyte-to-High-Density Lipoprotein Cholesterol Ratio (MHR), Granulocyte-to-High-Density Lipoprotein Cholesterol Ratio (GHR), Atherogenic Index of Plasma (AIP), and Triglyceride–Glucose Index (TyG), were measured during the first trimester (10–14 weeks of gestation), with the 75 g Oral Glucose Tolerance Test (OGTT) performed at 24–28 weeks serving as the gold standard. The results showed that using these markers related to glycolipid metabolism and inflammation alone could not adequately meet the clinical need for early GDM screening. The study further noted that no single early biomarker is currently capable of replacing the OGTT, and that the development of multi−indicator combined models represents a future direction. These findings provide empirical evidence for the limitations of relying on a single biomarker for early GDM risk stratification, and highlight the need to explore combined screening strategies with greater pathophysiological specificity (37).

A close pathophysiological link exists between Hcy and disorders of glucose and lipid metabolism, providing a rationale for combined screening strategies. Abnormal glucose metabolism (e.g., insulin resistance) and lipid metabolism disorders (e.g., dyslipidemia) are central to the pathogenesis of GDM and gestational hypertension. Hcy may interact with these metabolic disturbances by inducing oxidative stress, damaging vascular endothelium, and interfering with insulin signaling, thereby jointly contributing to the development and progression of pregnancy complications (10).

Numerous studies have confirmed an association between Hcy and key indicators of glucose metabolism. Serum levels of Hcy, AHSG, and CRP were significantly elevated in women with GDM compared to healthy controls, while 25-hydroxyvitamin D levels were significantly lower (38). The homeostatic model assessment of insulin resistance (HOMA-IR) showed a significant positive correlation with Hcy, total cholesterol (TC), and triglycerides (TG), suggesting that Hcy may contribute to GDM pathophysiology through interactions with glucose and lipid metabolic disturbances.

A recent review of novel biomarkers for GDM highlighted the correlation between Hcy and both insulin resistance and β-cell dysfunction, proposing Hcy as a candidate marker for early GDM screening (39). Supporting this, a subsequent study found that Hcy was positively correlated with HOMA-IR and negatively correlated with HOMA-β. Notably, they also found that in patients with GDM, Hcy was positively correlated with fasting plasma glucose (FPG), HbA1c, FINS, and cystatin C (40), providing new evidence that Hcy may indirectly contribute to insulin resistance through effects on renal or endothelial function. Another study measured serum levels of glycated serum protein (GSP), Hcy, and cystatin C in the third trimester and tracked perinatal outcomes. The findings confirmed that pregnant women with GDM had higher levels of GSP, Hcy, and cystatin C, and that higher levels of these three indicators were associated with a greater risk of adverse pregnancy outcomes. Moreover, the study found that combining these three markers could serve as an effective tool for predicting maternal and neonatal outcomes in GDM pregnancies (41). However, all of the above studies were single-center, small-sample investigations that did not adequately adjust for confounding factors, limiting the generalizability of their conclusions.

In a prospective cohort study of 150 diet-controlled GDM patients, Li et al. (42) demonstrated that fasting plasma glucose FPG and Hcy levels were significantly higher in the GDM group than in those with normal glucose tolerance. It was also observed that the apolipoprotein B/apolipoprotein A1 (apoB/apoA1) ratio was significantly elevated, whereas high-density lipoprotein cholesterol (HDL-C) and apoA1 levels were significantly reduced. However, no significant correlation was found between Hcy stratification and perinatal outcomes such as neonatal birth weight, indicating that the clinical significance of the Hcy-lipid relationship requires further clarification.

### 2.4 Hcy combined with nutritional and genetic indicators

Hcy levels are regulated by multiple factors, including modifiable nutritional factors such as folate and vitamin B12 intake, as well as non-modifiable genetic factors such as MTHFR gene polymorphisms (43, 44). Incorporating these upstream indicators into risk assessment could help achieve etiological subtyping and provide a basis for individualized intervention (10).

#### 2.4.1 Nutritional indicators: folic acid and vitamin B12

Numerous studies have demonstrated that nutritional factors are negatively correlated with Hcy levels (45).Tripathi et al. (46) demonstrated that folic acid and vitamin B12 supplementation effectively reduces circulating Hcy levels by promoting its conversion to methionine via the remethylation pathway, thereby preventing protein homocysteinylation and restoring cellular homeostasis. A cross-sectional study of 102 women in the second trimester (24–28 weeks) reported a significant inverse relationship between Hcy and both vitamin B12 and folic acid levels (P < 0.001), underscoring the importance of adequate supplementation for maintaining physiological Hcy concentrations (47).

Large-scale studies have further elucidated the clinical implications of this relationship. An investigation of 3,196 primiparas demonstrated that folic acid supplementation was associated with higher serum folic acid and vitamin B12 concentrations, reduced uterine artery resistance index (UtA-RI), and increased neonatal birth weight (48).

Findings from Chinese cohorts further corroborate these conclusions. In a large study of 14,178 couples preparing for pregnancy in Shanghai, China, Li et al. (49) demonstrated a statistically significant negative correlation between Hcy levels and red blood cell folate, serum folate, and vitamin B12 concentrations. Notably, the prevalence of abnormalities in these nutritional indicators was significantly higher in males than in females, with HHcy detected in 22.1% of men compared to only 2.5% of women. This gender disparity is clinically significant, given that the father’s folate status likewise influences sperm quality and the health of the offspring.

Above all, incorporating Hcy, folate, and vitamin B12 testing into preconception screening—alongside simultaneous evaluation and intervention for both partners—may help mitigate the risk of pregnancy complications originating from nutritional or genetic predispositions. While nutritional factors represent modifiable determinants of Hcy levels, genetic variants—particularly polymorphisms in the MTHFR gene—constitute non-modifiable risk factors that interact with nutritional status. The following section examines the role of genetic indicators in Hcy-related pregnancy risk assessment.

#### 2.4.2 Genetic indicators: MTHFR gene polymorphisms

At the genetic level, the MTHFR C677T and A1298C polymorphisms are the most extensively studied variants in one-carbon metabolism research. As noted in a review, MTHFR mutations are associated with elevated total Hcy levels, an effect that is particularly pronounced in pregnant women with inadequate folic acid intake (10).Zhao et al. (43)further explored the relationship between genotype, Hcy levels, and pregnancy outcomes in a retrospective analysis of 118 pregnant women. Carriers of the MTHFR 677TT and MTRR 66GG genotypes had a significantly increased risk of adverse pregnancy outcomes (95% CI: 2.881-5.942 and 1.427-3.809, respectively; P < 0.001). These carriers also showed a trend toward elevated Hcy levels and decreased vitamin B12 and red blood cell folate concentrations. In contrast, carriers of the MTRR 66AG genotype exhibited a protective effect. These findings support the hypothesis that genetic variations mediate pregnancy risk by modulating Hcy metabolism and vitamin levels.

#### 2.4.3 Gene–nutrition interaction and the value of combined screening

The influence of genetic factors on Hcy levels can be modulated by nutritional intervention. Mandić et al. (47) studied 102 pregnant women and found that despite high carrier rates of MTHFR C677T and A1298C polymorphisms (56.9% and 87.2%, respectively), there was no significant association between genotype and serum Hcy levels. The authors hypothesized that routine folic acid and vitamin B12 supplementation during pregnancy may compensate for mutation-related reductions in enzyme activity. This finding underscores the central role of gene–nutrition interactions in Hcy regulation.

Importantly, assessing genetic risk without considering nutritional status may lead to inaccurate risk stratification. Conversely, focusing solely on nutritional indicators may overlook inherent metabolic capacity defects. In this context, Hcy serves as a “functional endpoint” of gene–nutrition interactions. Combining Hcy measurement with nutritional and genetic indicators for comprehensive assessment may more accurately identify high-risk pregnant populations. D’Souza et al. (50) further elaborated on this interaction from a developmental biology perspective. They proposed that the integrated methionine and folate cycles not only regulate Hcy metabolism but also support embryonic growth, proliferation, and development through methylation reactions and nucleotide synthesis. When maternal nutrient intake is inadequate or genetic polymorphisms reduce enzyme activity, Hcy metabolic efficiency declines, potentially increasing the risk of adverse outcomes such as neural tube defects (51).

## 3 Conclusion

Hcy, as a key biomarker of one-carbon metabolism and vascular endothelial function, is increasingly being incorporated into multi-marker screening strategies for pregnancy complication risk management. Despite its promising prospects, the predictive performance of Hcy combined with different indicators for pregnancy complications remains variable and even contradictory. Most current evidence comes from single-center, small-sample retrospective studies, and there is considerable heterogeneity across studies in terms of gestational age, ethnicity, detection methods, and outcome definitions. Furthermore, some parameter measurements have not yet been standardized, which limits the comparability and generalizability of the findings. Future large-scale, multi-center prospective studies are needed to establish standardized predictive models and reference ranges (10, 12).
